# iLoc-Euk: A Multi-Label Classifier for Predicting the Subcellular Localization of Singleplex and Multiplex Eukaryotic Proteins

**DOI:** 10.1371/journal.pone.0018258

**Published:** 2011-03-30

**Authors:** Kuo-Chen Chou, Zhi-Cheng Wu, Xuan Xiao

**Affiliations:** 1 Gordon Life Science Institute, San Diego, California, United States of America; 2 Computer Department, Jing-De-Zhen Ceramic Institute, Jing-De-Zhen, China; Kyushu Institute of Technology, Japan

## Abstract

Predicting protein subcellular localization is an important and difficult problem, particularly when query proteins may have the multiplex character, i.e., simultaneously exist at, or move between, two or more different subcellular location sites. Most of the existing protein subcellular location predictor can only be used to deal with the single-location or “singleplex” proteins. Actually, multiple-location or “multiplex” proteins should not be ignored because they usually posses some unique biological functions worthy of our special notice. By introducing the “multi-labeled learning” and “accumulation-layer scale”, a new predictor, called **iLoc-Euk**, has been developed that can be used to deal with the systems containing both singleplex and multiplex proteins. As a demonstration, the jackknife cross-validation was performed with **iLoc-Euk** on a benchmark dataset of eukaryotic proteins classified into the following 22 location sites: (1) acrosome, (2) cell membrane, (3) cell wall, (4) centriole, (5) chloroplast, (6) cyanelle, (7) cytoplasm, (8) cytoskeleton, (9) endoplasmic reticulum, (10) endosome, (11) extracellular, (12) Golgi apparatus, (13) hydrogenosome, (14) lysosome, (15) melanosome, (16) microsome (17) mitochondrion, (18) nucleus, (19) peroxisome, (20) spindle pole body, (21) synapse, and (22) vacuole, where none of proteins included has 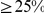 pairwise sequence identity to any other in a same subset. The overall success rate thus obtained by **iLoc-Euk** was 79%, which is significantly higher than that by any of the existing predictors that also have the capacity to deal with such a complicated and stringent system. As a user-friendly web-server, **iLoc-Euk** is freely accessible to the public at the web-site http://icpr.jci.edu.cn/bioinfo/iLoc-Euk. It is anticipated that **iLoc-Euk** may become a useful bioinformatics tool for Molecular Cell Biology, Proteomics, System Biology, and Drug Development Also, its novel approach will further stimulate the development of predicting other protein attributes.

## Introduction

Knowledge of the subcellular location of proteins is important as can be viewed from the following four aspects. (1) It can provide useful insights or clues about their functions; particularly, one of the fundamental goals in cell biology and proteomics is to identify the functions of proteins in the context of compartments that organize them in the cellular environment. (2) It can indicate how and in what kind of cellular environments the proteins interact with each other and with other molecules; this is especially important for the in-depth study of protein-protein interaction (PPI), one of the currently hot topics in proteomics. (3) It can help our understanding of the intricate pathways that regulate biological processes at the cellular level [1], [2] and hence it is indispensable for many studies in system biology. (4) It is very useful for identifying and prioritizing drug targets [3] during the process of drug development.

Although the knowledge of protein subcellular localization can be acquired by conducting various biochemical experiments, it is both time-consuming and costly by relying on doing experiments alone. Particularly, recent advances in large-scale genome sequencing have generated a huge number of protein sequences. For example, in 1986 the Swiss-Prot [4] database contained only 3,939 protein sequence entries, but now the number has jumped to 521,016 according to the release 2010_10 on 05-Oct-2010 by the UniProtKB/Swiss-Prot at http://www.expasy.org/sprot/relnotes/relstat.html; meaning that the number of protein sequence entries now is more than 132 times the number from about 24 years ago.

Facing the avalanche of protein sequences generated in the post-genomic age, it is highly desired to develop computational methods for timely and effectively identifying various biological features for newly found proteins [5], [6], [7], [8], particularly to develop user-friendly web-servers in this regard [9], [10]. In this study, we are to focus on the topic of protein subcellular localization.

Actually, the problem of predicting protein subcellular localization is somewhat reminiscent of the efforts by many previous investigators because during the past 19 years or so, a series of methods have been developed on this topic (see, e.g., [11], [12], [13], [14], [15], [16], [17], [18], [19], [20], [21], [22], [23], [24] as well as a long list of references cited in two comprehensive review articles [25], [26]). These methods each had their own advantages and indeed played a role in stimulating the development of this area although they also each had their own limitations.

The development of protein subcellular localization has generally followed two trends. One is to extract more useful information from protein sequences via different approaches or models, such as from the model of targeting or leader sequences [11], to the amino acid composition [13], [27], to the amino acid pair composition [28], to the various modes [21], [29], [30], [31], [32], [33], [34], [35], [36] of pseudo amino acid composition [37], and to the higher-level forms of pseudo amino acid composition by incorporating the functional domain information [38], gene ontology information [39], and sequential evolution information [40]. The other trend is to enhance the power of practical application by enlarging the coverage scope, such as from covering only 2 subcellular location sites [12], to 5 location sites [13], to 12 location sites [14], [28], and to 22 location sites [40].

Most of these existing methods were established based on the assumption that a protein resides at one, and only one, subcellular location (see, e.g., [13], [15], [28], [41], [42], [43], [44]). Such an assumption is valid only for the single-location or “singleplex” proteins but not for multiple-location or “multiplex” proteins that may simultaneously reside at, or move between, two or more different subcellular locations. Proteins with multiple location sites or dynamic feature of this kind are particularly interesting because they may have some unique biological functions worthy of our special notice [2], [3]. Particularly, as pointed out by Millar et al. [45], recent evidences have indicated that an increasing number of proteins have multiple locations in the cell.

Recently, a powerful predictor, called **Euk-mPLoc 2.0** [40] was developed that can be used to predict the subcellular localization of eukaryotic proteins among their 22 location sites in which some of the proteins may belong to two and more subcellular locations. However, **Euk-mPLoc 2.0** has the following shortcomings. (**1**) Only the integer numbers 0 and 1 were used to reflect the GO (gene ontology) [46], [47] information in formulating protein samples; this might cause some information lost and limit the prediction quality. (**2**) It was through an optimal threshold factor 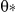 to control the prediction of multiple locations (see Eq.48 of [26]); it would be more natural if we could find a more intuitive approach to deal with such a problem. (**3**) Although a web-server for **Euk-mPLoc** has been established at http://www.csbio.sjtu.edu.cn/bioinf/euk-multi-2/, only one query protein sequence at a time is allowed when using the web-server to conduct prediction; for the convenience of users in handling many query protein sequences, such a rigid limit should be improved.

The present study was initiated in an attempt to develop a new and more powerful predictor by addressing the above three problems.

## Methods

Given a query protein sequence 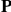 as formulated by 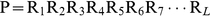
(1)where 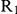 represents the 1^st^ residue of the protein 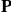, 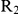
the 2^nd^ residue, …, 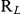 the 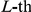 residue, and they each belong to one of the 20 native amino acids. How can we use its sequence information to predict which subcellular location(s) the protein 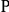 belongs to? The most straightforward method to address this problem is to use the sequence-similarity-search-based tools, such as BLAST [48], [49], to search protein database for those proteins with high sequence similarity to the query protein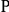. Subsequently, the subcellular location annotations of the proteins thus found are used to deduce the subcellular location(s) of 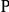. Unfortunately, this kind of straightforward and intuitive approach failed to work when the query protein 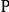 did not have significant sequence similarity to any location-known proteins.

Thus, various non-sequential or discrete models to represent protein samples were proposed in hopes to establish some sort of correlation or cluster manner through which the prediction could be more effectively carried out.

The simplest discrete model used to represent a protein sample is its amino acid (AA) composition or AAC [50]. According to the AAC-discrete model, the protein 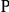 of **Eq.1** can be formulated by [51]

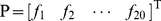
(2)where 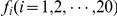 are the normalized occurrence frequencies of the 20 native amino acids in protein 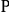, and 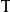 the transposing operator. Many methods for predicting protein subcellular localization were based on the AAC-discrete model (see, e.g., [12], [13],[14],[27] ). However, as we can see from **Eq.2**, if using the ACC model to represent the protein 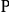, all its sequence-order effects would be lost, and hence the prediction quality might be limited.

To avoid completely lose the sequence-order information, the pseudo amino acid composition (PseAAC) was proposed to represent the sample of a protein, as formulated by [37]

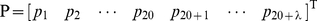
(3)where the first 20 elements are associated with the 20 amino acid components of the protein, while the additional 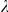 factors are used to incorporate some sequence-order information via a series of rank-different correlation factors along a protein chain.

Actually, the PseAAC for a protein 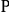 can be generally formulated as



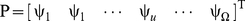
(4)where the subscript 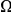 is an integer, and its value as well as the components 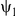, 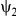, … will depend on how to extract the desired information from the amino acid sequence of 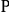 (cf. **Eq.1**). The form of **Eq.4** can cover the PseAAC as originally formulated in [37]; ie, when
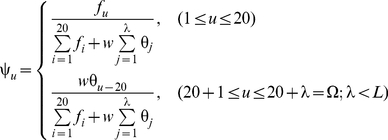
(5)we immediately obtain the formulation of PseAAC as originally given in [37], where the meanings for 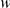, 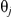, and 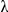 were clearly elaborated and hence there is no need to repeat here.

To develop a powerful method for statistically predicting protein subcellular localization, one of the most important things is to find a formulation to reflect the core and essential features of protein samples that are closely correlated with their subcellular localization. However, this is by no means an easy thing to do because this kind of features is usually deeply hidden or “buried” in piles of complicated sequences. To deal with this problem, let us consider the following approaches via the general form of PseAAC (**Eq.4**).

### 1. GO (Gene Ontology) Formulation

GO database [46] was established according to the molecular function, biological process, and cellular component. Accordingly, protein samples defined in a GO database space would be clustered in a way better reflecting their subcellular locations [26], [52]. However, in order to incorporate more information, instead of only using 0 and 1 elements as done in [40], here let us use a different approach as described below.

#### Step 1

Compression and reorganization of the existing GO numbers. The GO database (version 740 released 30 July 2009) contains many GO numbers. However, these numbers do not increase successively and orderly. For easier handling, some reorganization and compression procedure was taken to renumber them. For example, after such a procedure, the original GO numbers GO:0000001, GO:0000002, GO:0000003, GO:0000009, GO:00000011, GO:0000012, GO:0000015, …, GO:0090204 would become GO_compress: 0000001, GO_compress: 0000002, GO_compress: 0000003, GO_compress: 0000004, GO_compress: 0000005, GO_compress: 0000006, GO_compress: 0000007, ……, GO_compress: 0011118, respectively. The GO database obtained thru such a treatment is called GO_compress database, which contains 11,118 numbers increasing successively from 1 to the last one.

#### Step 2

Using **Eq.4** with 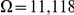, the protein 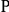 can be formulated as
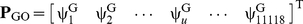
(6)where 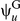 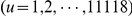
are defined via the following steps.

#### Step 3

Use BLAST [53] to search the homologous proteins of the protein 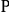 from the Swiss-Prot database (version 55.3), with the expect value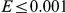 for the BLAST parameter.****

#### Step 4

Those proteins which have 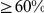 pairwise sequence identity with the protein 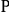 are collected into a set, 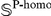, called the “homology set” of 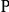. All the elements in 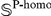 can be deemed as the “representative proteins” of 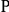, sharing some similar attributes such as structural conformations and biological functions [54], [55], [56]. Because they were retrieved from the Swiss-Prot database, these representative proteins must each have their own accession numbers.

#### Step 5

Search the GO database at http://www.ebi.ac.uk/GOA/ to find the corresponding GO number(s) [57] for each of the accession numbers collected in Step 4, followed by converting the GO numbers thus obtained to their GO_compress numbers as described in Step 1. (Note that the relationships between the UniProtKB/Swiss-Port protein entries and the GO numbers may be one-to-many, ‘‘reflecting the biological reality that a particular protein may function in several processes, contain domains that carry out diverse molecular functions, and participate in multiple alternative interactions with other proteins, organelles or locations in the cell’’ [46]. For example, the Uni-ProtKB/Swiss-Prot protein entry ‘‘P01040’’ corresponds to three GO numbers, i.e., ‘‘GO:0004866’’, ‘‘GO:0004869’’, and ‘‘GO:0005622’’).

#### Step 6

Thus, the elements in **Eq.6** is given by
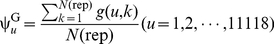
(7)where 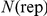 is the number of representative proteins in 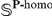, and 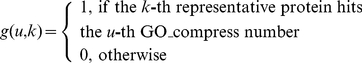
(8)


As we can see from **Eq.7**, the GO formulation derived from the above steps consists of 11,118 real numbers rather than only the elements 0 and 1 as in the GO formulation adopted in [40].

Note that the GO formulation of **Eq.6** may become a naught vector or meaningless under any of the following situations: (**1**) the protein 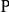 does not have significant homology to any protein in the Swiss-Prot database, i.e., 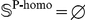 meaning the homology set 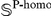
is an empty one; (**2**)its representative proteins do not contain any useful GO information for statistical prediction based on a given training dataset.

Under such a circumstance, let us consider using the sequential evolution formulation to represent the protein 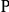, as described below.

### 2. SeqEvo (Sequential Evolution) Formulation

Biology is a natural science with historic dimension. All biological species have developed continuously starting out from a very limited number of ancestral species. It is true for protein sequence as well [56]. Their evolution involves changes of single residues, insertions and deletions of several residues [58], gene doubling, and gene fusion. With these changes accumulated for a long period of time, many similarities between initial and resultant amino acid sequences are gradually eliminated, but the corresponding proteins may still share many common attributes, such as having basically the same biological function and residing in a same subcellular location.

To incorporate the sequential evolution information into the PseAAC of **Eq.4**, here let us use the information of the PSSM (Position-Specific Scoring Matrix) [53], as described below.

#### Step 1

According to [53], the sequential evolution information of protein 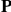 can be expressed by a 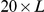 matrix as given by 
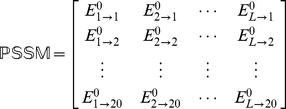
(9)where 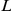 is the length of 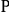 (counted in the total number of its constituent amino acids as shown in **Eq.1**), 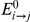
represents the score of the amino acid residue in the 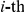 position of the protein sequence being changed to amino acid type 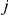 during the evolutionary process. Here, the numerical codes 1, 2, …, 20 are used to denote the 20 native amino acid types according to the alphabetical order of their single character codes. The 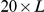 scores in **Eq.9** were generated by using PSI-BLAST [53] to search the UniProtKB/Swiss-Prot database (Release 2010_04 of 23-Mar-2010) through three iterations with 0001 as the 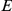-value cutoff for multiple sequence alignment against the sequence of the protein 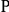.

#### Step 2

Use the elements in
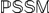
of Eq.9 to define a new matrix 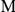 as formulated by
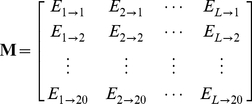
(10)with 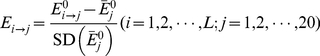
(11)where
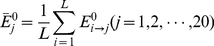
(12)is the mean for 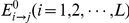 and
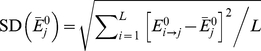
(13)is the corresponding standard deviation.

#### Step 3

Introduce a new matrix generated by multiplying 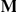 with its transpose matrix 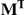; i.e.,
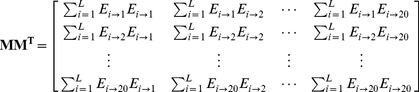
(14)which contains 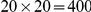 elements. Since 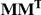 is a symmetric matrix, we only need the information of its 210 elements, of which 20 are the diagonal elements and 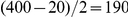 are the lower triangular elements, to formulate the protein 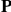; i.e., the general PseAAC form of **Eq.4** can now be formulated as
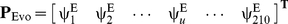
(15)where the components 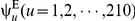 are respectively taken from the 210 diagonal and lower triangular elements of **Eq.14** by following a given order, say from left to right and from the 1^st^ row to the last as illustrated by following equation 
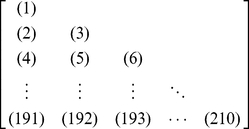
(16)where the numbers in parentheses indicate the order of elements taken from **Eq.14** for **Eq.15**.

### 3. The Self-consistency Formulation Principle

Regardless of using which formulation to represent protein samples, the following self-consistency principle must be observed during the course of prediction: if the query protein 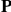 was defined in the form of 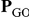 (see **Eq.6**), then all the protein samples used to train the prediction engine should also be expressed in the GO formulation; if the query protein was defined in the form of 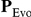 (see **Eq.15**), then all the training data should be expressed in the SeqEvo formulation as well.

Below, let us consider the algorithm or operation engine for conducting the prediction.

### 4. Multi-Label KNN (K-Nearest Neighbor) Classifier

In this study, let us introduce a novel classifier, called the multi-label KNN or abbreviated as ML-KNN classifier, to predict the subcellular localization for the systems that contain both single-location and multiple-location proteins.

**Figure 1:**
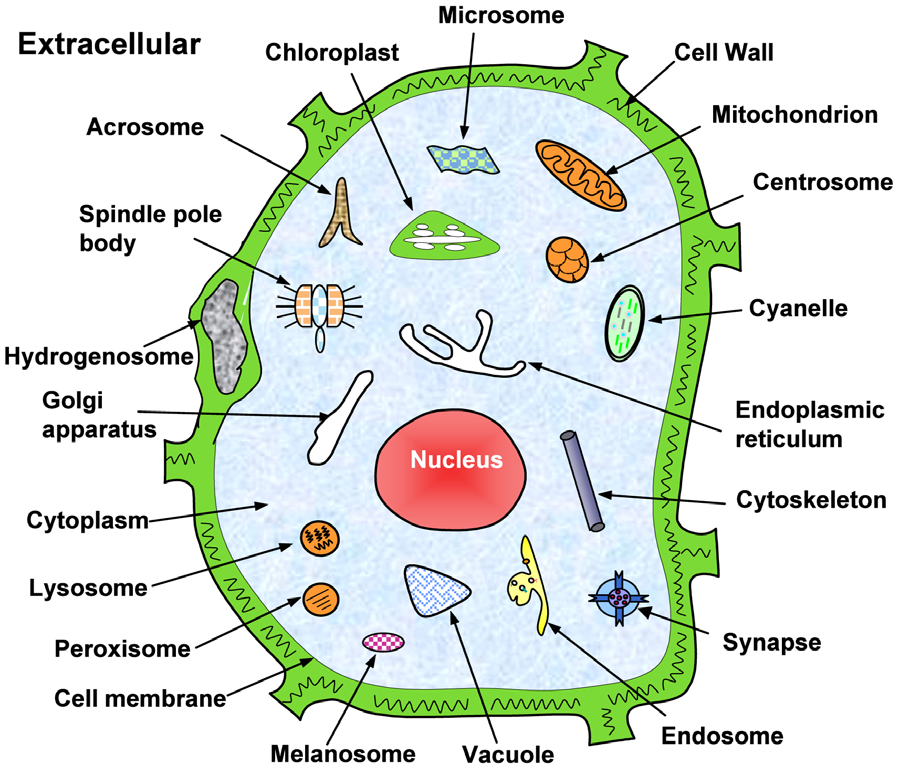
Illustration to show the 22 subcellular locations of eukaryotic proteins. The 22 locations are: (1) acrosome, (2) cell membrane, (3) cell wall, (4) centriole, (5) chloroplast, (6) cyanelle, (7) cytoplasm, (8) cytoskeleton, (9) endoplasmic reticulum, (10) endosome, (11) extracellular, (12) Golgi apparatus, (13) hydrogenosome, (14) lysosome, (15) melanosome, (16) microsome (17) mitochondria, (18) nucleus, (19) peroxisome, (20) spindle pole body, (21) synapse, and (22) vacuole. Adapted from [73] with permission.



Without losing generality, let us consider a system or dataset 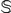 that contains 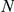 eukaryotic proteins classified into 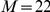 subcellular location sites (**Fig. 1**); i.e.,

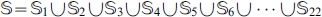
(17)where 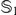 represents the subset for the subcellular location of “acrosome”, 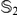 for “cell membrane”, 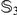 for “cell wall”, and so forth (cf **Table 1**); while 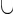 represents the symbol for “union” in the set theory. For convenience, hereafter let us just use the subscripts of **Eq.17** as the codes of the 22 location sites; i.e., “1” for “acrosome”, “2” for “cell membrane”, “3” for “cell wall”, and so forth (**Table 2**).

**Table 1:** A system or dataset 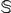 that contains 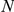 eukaryotic proteins classified into 22 subcellular location sites (cf. Eq.17), where the 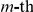 site or subset 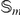 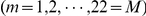 contains 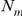 proteins. Note that since a protein may belong to more than one subcellular location, we generally have 
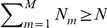
.

Subset ^a^	Subcellular location	Number of proteins
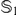	Acrosome	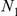
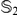	Cell membrane	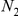
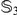	Cell wall	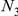
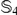	Centrosome	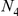
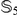	Chloroplast	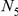
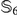	Cyanelle	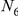
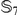	Cytoplasm	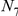
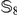	Cytoskeleton	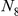
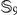	Endoplasmic reticulum	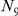
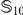	Endosome	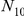
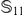	Extracellular	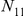
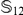	Golgi apparatus	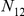
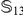	Hydrogenosome	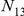
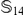	Lysosome	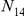
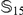	Melanosome	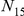
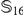	Microsome	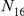
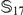	Mitochondrion	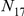
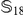	Nucleus	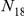
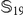	Peroxisome	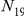
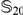	Spindle pole body	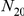
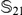	Synapse	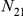
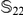	Vacuole	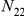

**Table 2:** A comparison of **iLoc-Euk** with **Euk-mPLoc 2.0** [40] using the jackknife cross-validation test on the benchmark dataset taken from the Online Supporting Information S1 of [40].

Code	Subcellular location site	Success rate by jackknife test	
		Euk-mPLoc 2.0 a	iLoc-Euk b
1	Acrosome	1/14 = 7.14%	1/14 = 7.14%
2	Cell membrane	452/697 = 64.85%	561/697 = 80.49%
3	Cell wall	6/49 = 12.24%	8/49 = 16.33%
4	Centrosome	22/96 = 22.92%	67/96 = 69.79%
5	Chloroplast	318/385 = 82.60%	338/385 = 87.79%
6	Cyanelle	47/79 = 59.49%	51/79 = 64.56%
7	Cytoplasm	1418/2186 = 64.87%	1677/2186 = 76.72%
8	Cytoskeleton	44/139 = 31.65%	38/139 = 27.34%
9	Endoplasmic reticulum	348/457 = 76.15%	407/457 = 89.06%
10	Endosome	2/41 = 4.88%	3/41 = 7.32%
11	Extracell	858/1048 = 81.87%	948/1048 = 90.46%
12	Golgi apparatus	56/254 = 22.05%	161/254 = 63.39%
13	Hydrogenosome	2/10 = 20.00%	0/10 = 0.00%
14	Lysosome	26/57 = 45.61%	18/57 = 31.58%
15	Melanosome	0/47 = 0.00%	1/47 = 2.13%
16	Microsome	1/13 = 7.69%	0/13 = 0.00%
17	Mitochondrion	427/610 = 70.00%	470/610 = 77.05%
18	Nucleus	1501/2320 = 64.70%	2040/2320 = 87.93%
19	Peroxisome	56/110 = 50.91%	60/110 = 54.55%
20	Spindle pole body	23/68 = 33.82%	45/68 = 66.18%
21	Synapse	0/47 = 0.00%	18/47 = 38.30%
22	Vacuole	101/170 = 59.41%	122/170 = 71.76%
Overall		5709/8897 = **64.17%** c	7034/8897 = **79.06%** c

The dataset contains 7,766 different eukaryotic protein sequences covering 22 location sites where none of the proteins included has 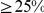 pairwise sequence identity to any other in a same location.

a The predictor from [40].

b The predictor proposed in this paper.

c Note that instead of 7,766 (the number of total different proteins), here we use 8,897 (the number of total different virtual proteins) for the denominator. This is because some proteins may have two or more location sites. As for the definition of “virtual protein”, see Eqs.2–3 of [40] and the relevant explanation there.

Suppose 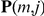 is the
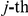
protein in the 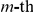
subset 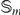 of 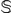 (**Eq.17**). Thus, we have 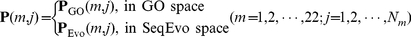
(18)where 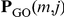 and 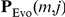 have the same forms as 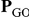
(**Eq.6**), and 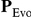
(**Eq.15**), respectively; the only difference is that the corresponding constituent elements are derived from the amino acid sequence of 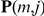 instead of 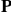.

In sequence analysis, there are many different scales to define the distance between two proteins, such as Euclidean distance, Hamming distance [59], and Mahalanobis distance [51], [60], [61]. In [40], the distance between 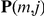 and 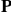 was defined by 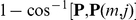. However, we found that when the GO descriptor was formulated with real numbers, better results would be obtained by using the Euclidean metric; i.e., the distance between 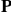 and 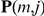 is defined here by
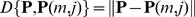
(19)where 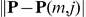 represents the module of the vector difference between 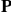 and 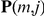 in the Euclidean space. According to **Eq.19**, when 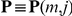 we have 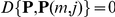, indicating the distance between these two protein sequences is zero and hence they have perfect or 100% similarity.

Suppose 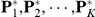 are the *K* nearest neighbor proteins to the protein 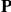 that forms a set denoted by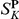, which is a subset of 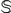; i.e.,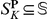 Based on the *K* nearest neighbor proteins in 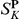, let us define an accumulation-layer (AL) scale, given by
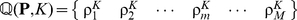
(20)where 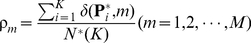
(21)where 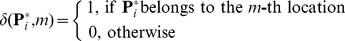
(22)and 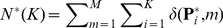
(23)Note that 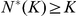 because a protein may belong to one or more subcellular location sites in the current system.

Now, for a query protein 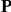, its subcellular location(s) will be predicted according to the following steps.

#### Step 1

The number of how many different subcellular locations it belongs to will be determined by its nearest neighbor protein in 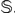 For example, suppose 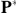 is the nearest protein to 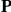 in 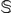. If 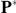 has only one subcellular location, then 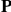 will also have only one location; if 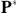 has two subcellular locations, then 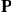 will also have two locations; and so forth. In general, if 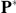 belongs to 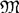 different location sites, then 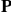 will be predicted to have the same number, 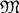, of subcellular locations as well, as can be formulated by 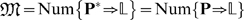
(24)where 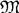 is an integer 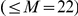, 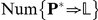 represents the number of different subcellular locations to which 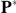 belongs, and so forth.

#### Step 2

However, the concrete location site(s) to which 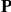 belongs will not be the same as 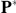 does, but determined by the element(s) in **Eq.20** that has (have) the highest score(s), as can be expressed by 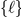, the subscript(s) of **Eq.17**. For example, if 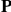 is found belonging to only one location 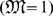 in Step 1, and the highest score in **Eq.20** is 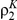, then 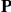 will be predicted as 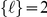 meaning that it belongs to 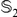 or resides at “cell membrane” (cf. **Table 1**). If 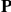 is found belonging to three locations 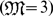 in Step 1, and the first three highest scores in **Eq.20** are 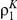, 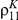, and 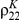, then 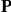 will be predicted as 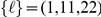 meaning that it belongs to 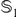,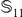 and 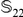 or resides simultaneously at “acrosome”, “extracellular”, and “vacuole”. And so forth. In other words, the concrete predicted subcellular location(s) can be formulated as
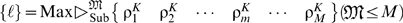
(25)where the operator “
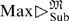
” means identifying the 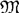 highest scores for the elements in the brackets right after it, followed by taking their 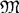 Subscripts.

**Figure 2:**
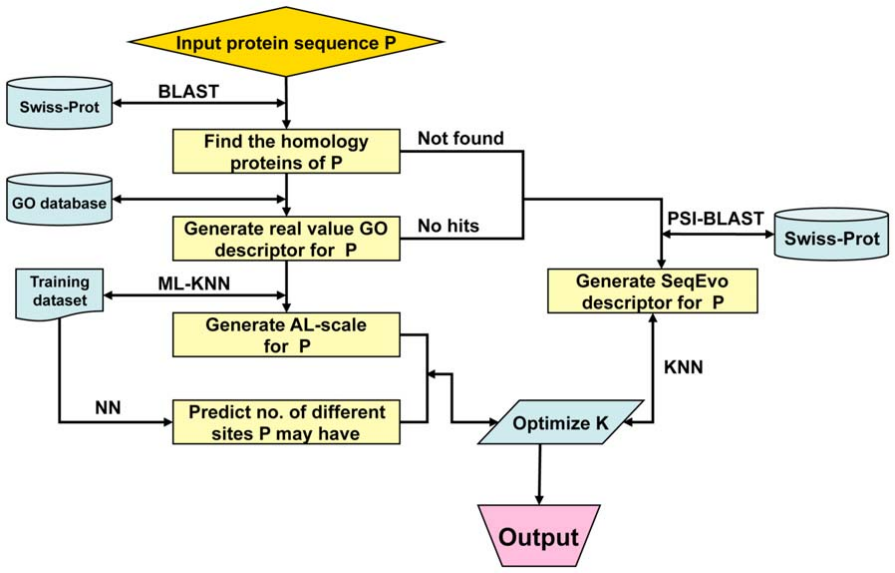
A flowchart to show the prediction process of iLoc-Euk.

The entire classifier thus established is called **iLoc-Euk**, which can be used to predict the subcellular localization of both singleplex and multiplex eukaryotic proteins. To provide an intuitive picture, a flowchart is provided in **Fig. 2** to illustrate the prediction process of **iLoc-Euk.**

### 5. Protocol Guide

For those who are interested in using the predictor but not its mathematical details, a web-server for **iLoc-Euk** was established. Below, let us give a step-by-step guide on how to use it to get the desired results.

#### Step 1

**Figure 3:**
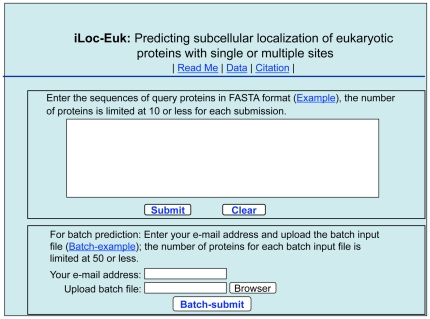
A semi-screenshot to show the top page of the iLoc-Euk web-server. Its website address is at http://icpr.jci.edu.cn/bioinfo/iLoc-Euk.

Open the web server at site http://icpr.jci.edu.cn/bioinfo/iLoc-Euk and you will see the top page of the predictor on your computer screen, as shown in **Fig. 3**. Click on the Read Me button to see a brief introduction about **iLoc-Euk** predictor and the caveat when using it.

#### Step 2

Either type or copy and paste the query protein sequence into the input box at the center of **Fig. 3**. The input sequence should be in the FASTA format. A sequence in FASTA format consists of a single initial line beginning with a greater-than symbol (“>”) in the first column, followed by lines of sequence data. The words right after the “>” symbol in the single initial line are optional and only used for the purpose of identification and description. All lines should be no longer than 120 characters and usually do not exceed 80 characters. The sequence ends if another line starting with a “>” appears; this indicates the start of another sequence. Example sequences in FASTA format can be seen by clicking on the Example button right above the input box. For more information about FASTA format, visit http://en.wikipedia.org/wiki/Fasta_format. Different with **Euk-mPLoc 2.0** [40], where only one query protein sequence is allowed as an input for each submission, now the maximum number of query proteins can be 10.

#### Step 3

**Figure 4:**
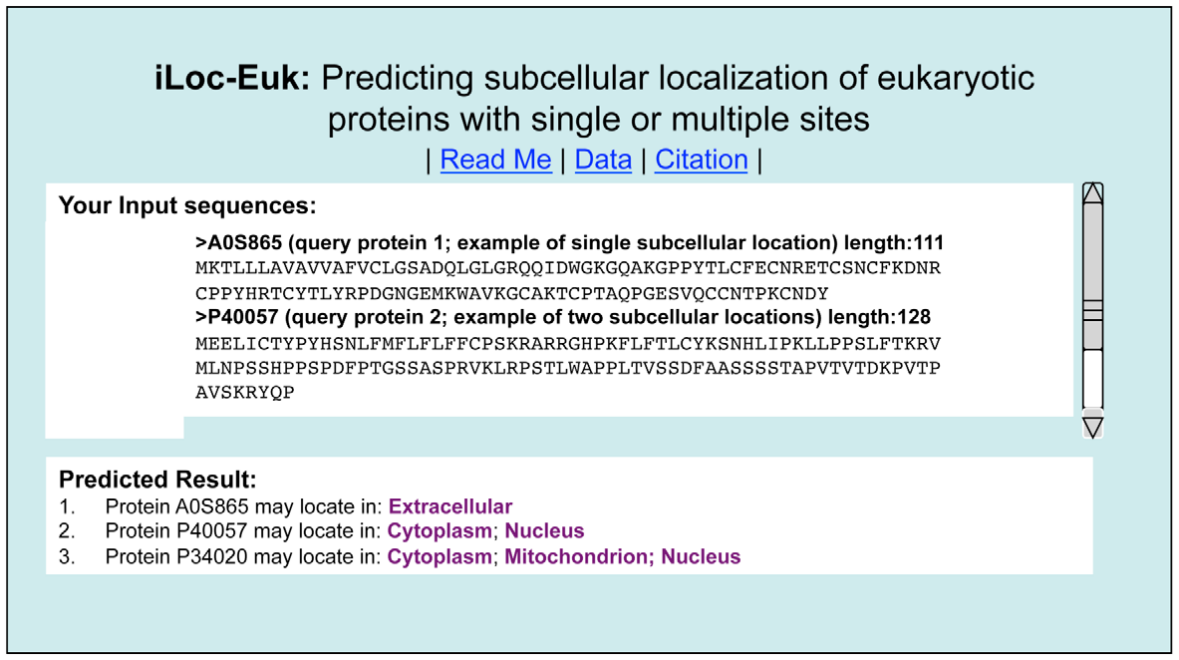
A semi-screenshot to show the output of iLoc-Euk. The input was taken from the three protein sequences listed in the Example window of the iLoc-Euk web-server (cf. Fig. 3).

Click on the Submit button to see the predicted result. For example, if you use the three query protein sequences in the Example window as the input, after clicking the Submit button, you will see **Fig. 4** shown on your screen, indicating that the predicted result for the 1^st^ query protein is “**Extracellular**”, that for the 2^nd^ one is “**Cytoplasm; Nucleus**”, and that for the 3^rd^ one is “**Cytoplasm; Mitochondrion; Nucleus**”. In other words, the 1^st^ query protein (A0S865) is a single-location one residing at “extracellular” only, the 2^nd^ one (P40057) can simultaneously occur in two different sites (“cytoplasm” and “nucleus”), and the 3^rd^ one (Q05043) can simultaneously occur in three different sites (“cytoplasm”, “mitochondrion”, and “nucleus”). All these results are fully consistent with the experimental observation as summarized in the Online Supporting Information S1 [40]. It takes about 10 seconds for the above computation before the predicted result appears on your computer screen; the more number of query proteins and longer of each sequence, the more time it is usually needed.

#### Step 4

As shown on the lower panel of **Fig. 3**, you may also choose the batch prediction by entering your e-mail address and your desired batch input file (in FASTA format) via the “Browse” button. To see the sample of batch input file, click on the button Batch-example The maximum number of the query proteins for each batch input file is 50. After clicking the button Batch-submit, you will see “Your batch job is under computation; once the results are available, you will be notified by e-mail.” Note that if you submit a batch input file from an Apple computer, although it looks like in the FASTA format, your input might change to non-FASTA format in the server end and cause errors. Under such a circumstance, the safest way is to submit your input file with a pdf format.

#### Step 5

Click on the Citation button to find the relevant papers that document the detailed development and algorithm of **iLoc-Euk.**

#### Step 6

Click on the Data button to download the benchmark datasets used to train and test the **iLoc-Euk** predictor .

#### Caveat

To obtain the predicted result with the expected success rate, the entire sequence of the query protein rather than its fragment should be used as an input. A sequence with less than 50 amino acid residues is generally deemed as a fragment. Also, if the query protein is known not one of the 22 locations as shown in **Fig. 1**, stop the prediction because the result thus obtained will not make any sense.

## Results and Discussion

In statistical prediction, it would be meaningless to simply say a success rate of a predictor without specifying what method and benchmark dataset were used to test its accuracy. As is well known, the following three methods are often used to examine the quality of a predictor: independent dataset test, subsampling test, and jackknife test [62]. Since independent dataset can be treated as a special case of subsampling test, one benchmark dataset is sufficient to serve all the three kinds of cross-validation. However, as demonstrated by Eq.1 of [63] and elucidated in [26], among the three cross-validation methods, the jackknife test is deemed the least arbitrary that can always yield a unique result for a given benchmark dataset and hence has been widely recognized and increasingly used to examine the power of various predictors (see, e.g., [17],[64],[65],[66],[67],[68],[69],[70],[71],[72]). Accordingly, the jackknife test will be used in this study to evaluate the power of **iLoc-Euk.**

However, even if using the jackknife approach for cross-validation, a same predictor may still generate obviously different success rates when tested by different benchmark datasets. This is because the more stringent of a benchmark dataset in excluding homologous and high similarity sequences, the more difficult for a predictor to achieve a high overall success rate [40]. Also, the more number of subsets (subcellular locations) a benchmark dataset covers, the more difficult to achieve a high overall success rate. This can be easily conceivable via the following consideration. Suppose a benchmark dataset consists of two subsets (subcellular locations) with each containing a same number of proteins. The overall success rate in identifying their attribute categories by random assignment would be 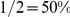. However, for a benchmark dataset consisting of 22 subsets (subcellular locations), the corresponding overall success rate by the random assignment would be only 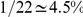.

In this study, the same benchmark dataset 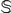 as investigated in [40] was adopted for demonstration. The dataset can be obtained from the Online Supporting Information S1 of [40]. It can also be directly downloaded from the web-site at http://www.csbio.sjtu.edu.cn/bioinf/euk-multi-2/Data.htm. The reasons we choose it as a benchmark dataset for the current study are as follows. (1) The dataset was constructed specialized for eukaryotic proteins and it can cover 22 subcellular location sites; compared with the other datasets in this area that only covered 5-10 subcellular locations, the coverage scope of the current dataset is much wider. (2) None of proteins included in the current benchmark dataset has 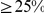 pairwise sequence identity to any other in a same subcellular location; compared with most of the other benchmark datasets in this area, the current one is much more stringent in excluding homology bias and redundancy. (3) It contains both singleplex and multiplex proteins and hence can be used to train and test a predictor developed aimed at being able to deal with proteins with both single and multiple location sites. (4) Using the current benchmark dataset will also make it more fair and easier to compare the new predictor with the existing one because the tested results by **Euk-mPLoc 2.0** on the current benchmark dataset have been well documented and reported is a recent paper [40].

The dataset 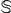 contains 7,766 different eukaryotic proteins, of which 6,687 belong to one subcellular location, 1,029 to two locations, 48 to three locations, and 2 to four locations.

For such a complicated dataset containing both single-location and multiple-location proteins distributed among 22 subcellular location sites, so far only two existing predictors, i.e., **Euk-mPLoc** [73] and **Euk-mPLoc 2.0** [40], had the capacity to deal with it. It was reported [40] that, when tested by the dataset 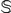, the overall jackknife success rate achieved by **Euk-mPLoc 2.0** was about 25% higher than that by **Euk-mPLoc**. Therefore, to demonstrate the power of the predictor proposed in this paper, it would be sufficient to just compare **iLoc-Euk** with **Euk-mPLoc 2.0** [40].

Listed in **Table 2** are the results obtained with **Euk-mPLoc 2.0** [40] and **iLoc-Euk** on the aforementioned benchmark dataset 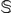 by the jackknife test. As we can see from **Table 2**, for such a stringent and complicated benchmark dataset, the overall success rate achieved by **iLoc-Euk** is over 79%, which is about 15% higher than that by **Euk-mPLoc 2.0**.

Note that during the course of the jackknife test by **Euk-mPLoc 2.0** and **iLoc-Euk**, the false positives (over-predictions) and false negatives (under-predictions) were also taken into account to reduce the scores in calculating the overall success rate. As for the detailed process of how to count the over-predictions and under-predictions for a system containing both single-location and multiple-location proteins, see Eqs.43–48 and Fig. 4 in a comprehensive review [26].

To provide a more intuitive and easier-to-understand measurement, let us introduce a new scale, the so-called “absolute true” success rate, to reflect the accuracy of a predictor, as defined by
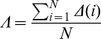
(26)where 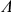 represents the absolute true rate, 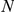 the number of total proteins investigated, and
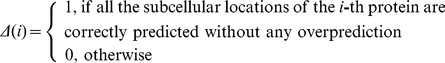
(27)According to the above definition, for a protein belonging to, say, three subcellular locations, if only two of the three are correctly predicted, or the predicted result contains a location not belonging to the three, the prediction score will be counted as 0. In other words, when and only when all the subcellular locations of a query protein are exactly predicted without any underprediction or overprediction, can the prediction be scored with 1. Therefore, the absolute true scale is much more strict and harsh than the scale used previously [26], [40] in measuring the success rate. However, even if using such a stringent criterion on the same benchmark dataset by the jackknife test, the overall absolute true success rate achieved by **iLoc-Euk** was 5535/7766 = 71.27%.

The reasons why **iLoc-Euk** can achieve higher success rates than **Euk-mPLoc 2.0** are as follows. (**1**)The GO formulation used to represent protein samples in **iLoc-Euk** is formed by the hit probabilities and hence contains more information than that in **Euk-mPLoc 2.0** [40] where only the number “0” or “1” was used regardless how many hits were found to the corresponding component in the GO formulation. (**2**) The accumulation-layer scale has been introduced in **iLoc-Euk** that is particularly useful and more natural for dealing with proteins having multiple subcellular locations.

Finally, it should be pointed out that although **iLoc-Euk** is more powerful than the existing predictors in identifying the subcellular locations of eukaryotic proteins, there is much room for further improvement in future studies. As shown in **Table 2**, the success rates by **iLoc-Euk** for proteins belonging to “hydrogenosome” and “microsome” locations are still very low. This is because of that, compared with the most of the other 20 location sites, the numbers of proteins in the two sites are not sufficiently large to train the prediction engine in a more effective way. It is anticipated that with more experimental data available for the two sites in the future, the situation will be improved and the anticipated success rates by **iLoc-Euk** will be further enhanced.

## Supporting Information

Supporting Information S1The benchmark dataset 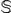 used in this study contains 7,766 different eukaryotic protein sequences classified into 22 subsets according to their subcellular locations. Of the 7,766 different proteins, 6,687 belong to one subcellular location, 1,029 to two locations, 48 to three locations, and 2 to four locations. Both the accession numbers and sequences are given. None of the proteins included has 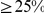 pairwise sequence identity to any other in the same subset. See **Table 1** and the relevant text of the paper for further explanation.(PDF)
